# Cell-specific effects of the sole *C. elegans* Daughterless/E protein homolog, HLH-2, on nervous system development

**DOI:** 10.1242/dev.201366

**Published:** 2023-01-03

**Authors:** Neda Masoudi, Ralf Schnabel, Eviatar Yemini, Eduardo Leyva-Díaz, Oliver Hobert

**Affiliations:** ^1^ Department of Biological Sciences, Columbia University, Howard Hughes Medical Institute, New York, NY 10027, USA; ^2^ Institute of Genetics, Technische Universität Braunschweig, 38106 Braunschweig, Germany; ^3^ University of Massachusetts, Department of Neurobiology, Worcester, MA 1605-2324, USA

**Keywords:** *Caenorhabditis elegans*, bHLH, Neurogenesis, HLH-2, Daughterless, E protein

## Abstract

Are there common mechanisms of neurogenesis used throughout an entire nervous system? We explored to what extent canonical proneural class I/II bHLH complexes are responsible for neurogenesis throughout the entire *Caenorhabditis elegans* nervous system. Distinct, lineage-specific proneural class II bHLH factors are generally thought to operate via interaction with a common, class I bHLH subunit, encoded by Daughterless in flies, the E proteins in vertebrates and HLH-2 in *C. elegans*. To eliminate function of all proneuronal class I/II bHLH complexes, we therefore genetically removed maternal and zygotic *hlh-2* gene activity. We observed broad effects on neurogenesis, but still detected normal neurogenesis in many distinct neuron-producing lineages of the central and peripheral nervous system. Moreover, we found that *hlh-2* selectively affects some aspects of neuron differentiation while leaving others unaffected. Although our studies confirm the function of proneuronal class I/II bHLH complexes in many different lineages throughout a nervous system, we conclude that their function is not universal, but rather restricted by lineage, cell type and components of differentiation programs affected.

## Teaser

**Summary:** This paper shows that canonical proneural bHLH complexes do not globally affect neurogenesis in *C. elegans* but have selective effects in specific linages on specific neuronal features.

## INTRODUCTION

**Fig. 1.**
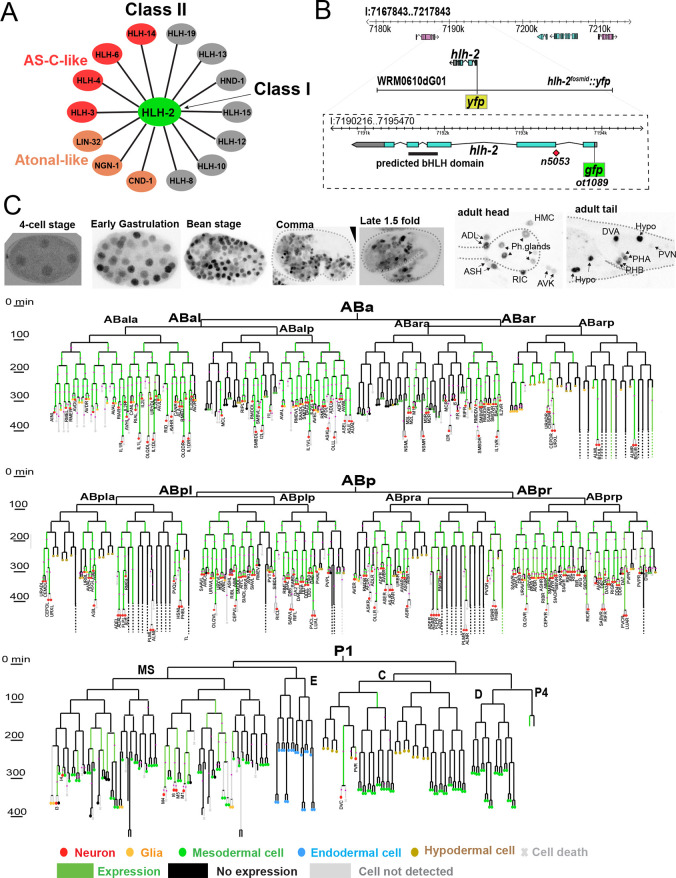
**Background for HLH-2 protein function and description of its expression pattern.** (A) Physical interaction of HLH-2 (class I) with class II proteins as determined by Grove et al. (2009). Proneural AS-C and Atonal homologs are color-coded. No homodimerization of class II protein was detected. (B) Schematic of gene structure, indicating mutation of G to A in the splice acceptor of second exon in *n5053* allele and reporter genes. The YFP box shows the position of fluorescent reporter in the fosmid reporter (*otIs502*) and the GFP box represent the insertion of *gfp*, upstream of ATG in the CRISPR/Cas9-engineered reporter allele (*ot1089*), both of which show similar expression patterns. (C) Representative images of *hlh-2* reporter allele expression throughout embryonic development (upper panel). The lower panels exhibit persistence of *hlh-2* signal in subset of neurons postembryonically. Dashed outlines indicate embryo shape. The lineage diagram indicates *hlh-2* fosmid expression (*otIs502*) in the AB and P lineages. The purple stars on the lineage diagrams indicate concordance with independent, semi-automated lineaging described by Ma et al. (2021), using our fosmid reporter construct.

Proneural basic helix-loop-helix (bHLH) transcription factors are phylogenetically conserved drivers of neurogenesis. Mutant analysis in flies and worms, as well as gain-of-function experiments in vertebrates, revealed that members of this family are both required and sufficient for initial steps of neurogenesis (reviewed by Jan and Jan, 1994; Hassan and Bellen, 2000; Bertrand et al., 2002; Wang and Baker, 2015; Baker and Brown, 2018). Proneural bHLH factors fall into two families: the Achaete Scute family, members of which include the vertebrate MASH genes and *Drosophila* AS-C genes, and the Atonal family, which includes fly Atonal and its vertebrate MATH orthologs, as well as *Drosophila* and vertebrate neurogenin and NeuroD proteins (Hassan and Bellen, 2000; Bertrand et al., 2002; Baker and Brown, 2018). Achaete Scute and Atonal family members are class II bHLH proteins that heterodimerize with a broadly expressed common class I bHLH protein (Massari and Murre, 2000) (Fig. 1A). As expected from the phenotype of their class II interaction partners, class I proteins, like fly Daughterless, also have proneural activity (Wang and Baker, 2015; Baker and Brown, 2018). Although proneural genes have been implicated in neurogenesis in many parts of invertebrate and vertebrate nervous systems, the extent to which canonical proneural class I/II bHLH complexes control neurogenesis throughout an entire nervous system has not been determined.

We sought to address this question in a nervous system-wide manner in the nematode *Caenorhabditis elegans.* The *C. elegans* genome codes for homologs of all genes classified as ‘proneural’ in other systems (Bertrand et al., 2002) (Fig. 1A). This includes three members of the Atonal family (a single Atonal ortholog, *lin-32*; a single neurogenin ortholog, *ngn-1*; a single NeuroD ortholog, *cnd-1*), as well as five members of the AS-C family (*hlh-3*, *hlh-4*, *hlh-6*, *hlh-14*, *hlh-19*) (Ledent et al., 2002; Simionato et al., 2007) (Fig. 1A). Proneural functions have been identified for several of these class II genes and, as expected, these proneural functions have been shown to also involve the sole *C. elegans* ortholog of the *Drosophila* class I bHLH heterodimerization partner *daughterless*, *hlh-2* (Zhao and Emmons, 1995; Krause et al., 1997; Portman and Emmons, 2000; Frank et al., 2003; Nakano et al., 2010; Poole et al., 2011; Luo and Horvitz, 2017; Masoudi et al., 2021), a notion further confirmed by biochemical interaction analysis (Grove et al., 2009) (Fig. 1A). However, a nervous system-wide analysis of proneural genes in neuronal fate induction has been missing. The specific advantages that *C. elegans* brings to this problem is its nervous system-wide perspective: all *C. elegans* neurons are precisely mapped out, its neuron number is limited (302 neurons in the hermaphrodite) and molecular markers exist for each individual neuron class, thereby allowing us to probe proneural bHLH function with single cell and nervous system-wide resolution.

Here, we provide a nervous system-wide view of canonical proneural class I/II bHLH complex activities by analyzing the effects of the removal of *hlh-2*. Based on the obligate heterodimer formation observed for all *C. elegans* proneural bHLH proteins (Grove et al., 2009) (Fig. 1A), the genetic removal of *hlh-2* is expected to disable the function of all proneural bHLH genes. This would address to what extent proneural bHLH genes can be made responsible for the generation of all neuronal cell types in *C. elegans.* A similar approach has not yet been taken in other organisms. In *Drosophila* larvae, Daughterless is required for the specification of many neurons of the peripheral nervous system (Caudy et al., 1988; Vaessin et al., 1994; Wang and Baker, 2015). However, this conclusion is tempered by the limited number of molecular markers examined. Moreover, the maternal contribution of Daughterless could not be removed without affecting reproduction, therefore leaving it unclear whether Daughterless may have broader roles in the differentiation of the CNS of the fly. There are three class I genes in vertebrates, *E12*/*E47*, *E2-2* (*TCF4*) and *HEB* (*TCF12*), but their function in neurogenesis, either in single or compound mutants, has not been comprehensively analyzed to date (Wang and Baker, 2015).

## RESULTS

### Expression pattern of GFP-tagged HLH-2 protein

Previous HLH-2 protein expression analysis, using antibody staining, showed transient expression in many parts of the developing embryo, as well as sustained expression in a few postembryonic cell types (Krause et al., 1997). However, the identity of many of the expressing cells remained unclear or tentative. We have used a chromosomally integrated fosmid reporter strain, *otIs502*, in which *hlh-2* is tagged at the N terminus with *yfp*, as well as a CRISPR/Cas9 engineered reporter allele, with an N-terminal *gfp* insertion (*ot1089*) to precisely delineate *hlh-2*-expressing cells (Fig. 1B). For embryonic cell identification, we used 4D microscopy in conjunction with Simi BioCell software to trace *hlh-2* expression during embryonic development (Schnabel et al., 1997). Consistent with antibody staining (Krause et al., 1997), we detected low levels of HLH-2::GFP at very early embryonic stages. Signals markedly increase at different time points in different lineages (Fig. 1C). Expression was eventually established throughout most of the developing embryo, including all neuron producing lineages (Fig. 1C). The only exception is the ABarapa lineage, which gives rise to four pharyngeal neurons (MCR, I1R, I2R, I5).

Expression of *hlh-2::gfp* in the nervous system is mostly transient. It becomes undetectable in the vast majority of postmitotic neurons toward the end of embryogenesis. Postembryonic expression is only observed in four sensory neuron classes (ADL, ASH, PHA and PHB) and four interneuron classes (RIC, AVK, DVA, PVN) (Fig. 1C). *hlh-2::gfp* is expressed in all the P neuroblasts, which give rise to ventral nerve cord motor neurons, at the L1 stage but subsequently fades.

Together with proneural class II bHLH expression patterns that we reported earlier (Masoudi et al., 2018, 2021), a complete picture of proneural bHLH expression patterns emerges. As summarized in Table S1, *hlh-2* shows the broadest expression throughout the embryonic nervous system, followed by the also very broadly expressed AS-C homolog *hlh-3*. The other known proneural AS-C homolog *hlh-14* is the next most broadly expressed bHLH. ATO superfamily members are more narrowly expressed than *hlh-14*. Among the ATO superfamily members, *ngn-1* and *cnd-1* are more broadly expressed than *lin-32.*

### Lineage analysis of maternal/zygotic *hlh-2* mutant animals

Unlike most of the other proneural class II bHLH genes, strong loss-of-function mutants of the class I gene *hlh-2* display completely penetrant embryonic lethality (Nakano et al., 2010). We sought to remove potential maternal contributions using an unstable transgenic array that contains the wild-type *hlh-2* locus (see Materials and Methods). We selected animals that had lost the array in the germline-producing lineage of their mothers. Such *hlh-2* maternal/zygotic mutants (henceforth called *hlh-2^m/z^* mutants) indeed display a stronger phenotype than zygotic *hlh-2* mutants derived from a heterozygous mother (see Materials and Methods). We set out to perform a cellular lineage analysis of *hlh-2^m/z^* animals using 4D lineaging using Simi BioCell software (Schnabel et al., 1997). We focused on the AB lineage from which most of the 221 embryonically born neurons derive (several of the few pharyngeal neurons derived from the MS lineage were previously also found to require proneural bHLH factors, including *hlh-*2, for proper specification) (Nakano et al., 2010; Luo and Horvitz, 2017).

**Fig. 2.**
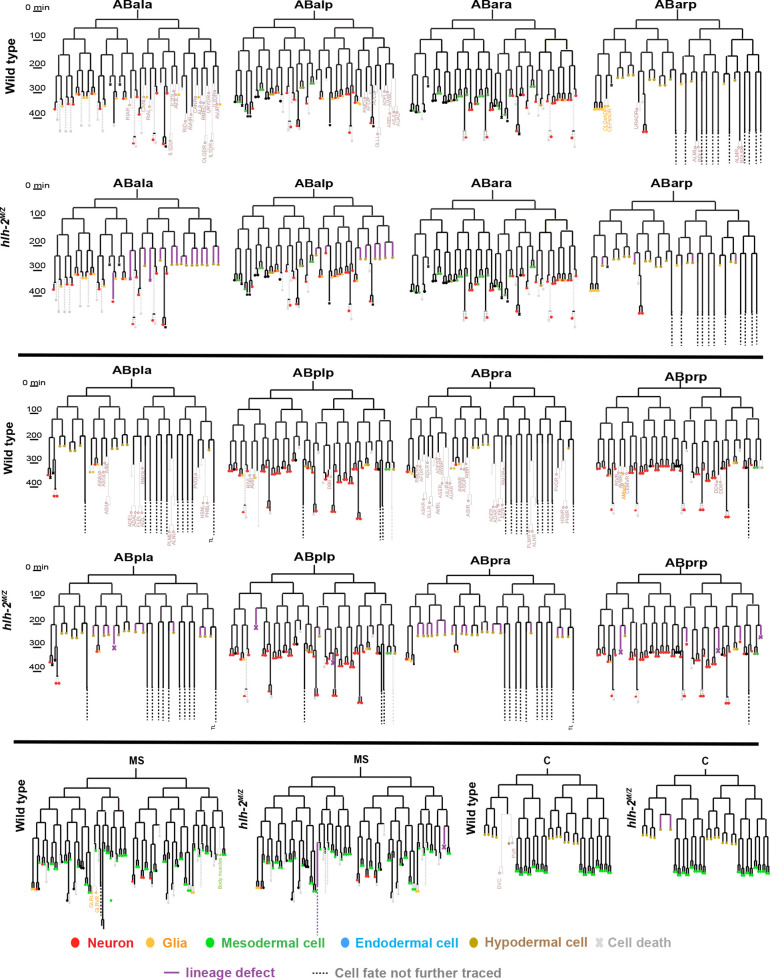
**Lineage analysis of *hlh-2^m/z^* mutant animals.** ABa, ABp, MS and C lineages are shown for detailed side-to-side comparison of wild type with *hlh-2(n5053)* mosaic progeny. The OH16873 strain was used for mosaic analysis. To eliminate any maternal contribution, hermaphrodites exhibiting the mosaic pattern that indicates loss of rescuing array in P2 lineage (*hlh-2^m/z^* mutant animals) were picked and their progeny were lineaged using Simi BioCell. Each lineage was analyzed for timing and pattern of divisions, cell positions and potential cell fates based on morphological features using DIC imaging. The lineages affected are highlighted in the wild-type panels with pale colors for ease of comparison. The inferred cellular fate is shown in the *hlh-2* panel, highlighted with purple lines, below or beside every respective wild-type lineage. See Tables S2 and S3 for alternative presentations of these lineage transformation defects.

We found that 68 neurons are not born in *hlh-2^m/z^* mutants; instead, their mother or grandmother is transformed to hypodermal cell fate. Like conventional hypodermal cells, these *de novo* hypodermal cells stop dividing after the ninth division, migrate to the surface of the embryo and do not display the speckled nuclei that are characteristic of neuronal nuclei. In addition, nine cells that are normally destined to become a neuron instead undergo programmed cell death (apoptosis) in *hlh-2^m/z^* embryos, exhibiting button-like condensed nuclei (Fig. 2; Tables S2, S3). Furthermore, the nuclei of the glia-like GLR cells in the mutant look more like neuronal nuclei, indicating that these cells might have also switched their fate in *hlh-2* mutants. Other notable fate switches include the transformation of the tail spike cell and some of the glial socket and sheath cells to hypodermal fate. We also observed an additional 23 hypodermal cells in place of cells normally destined to die by apoptosis. Among cells fated to go through apoptosis we have also observed that a few instead exhibited neuronal features (speckled nuclei) and some that even went through an additional division (Fig. 2; Tables S2, S3).

Taken together, our lineage analysis indicates that 122 of the 221 embryonically born cells fated to become neurons appear to be generated normally, as assessed by nuclear neuronal morphology, while 78 of the 221 embryonically born neurons are not generated and instead adopt alternative fates (either hypodermal or apoptosis) (Fig. 2; Tables S2, S3). The remaining 21 neurons (221 in total, minus 122 neuron-like, minus 78 transformed) could not be lineage-traced or their morphological identity is ambiguous.

### Examination of hypodermal and neuronal fate markers in *hlh-2* mutants

**Fig. 3.**
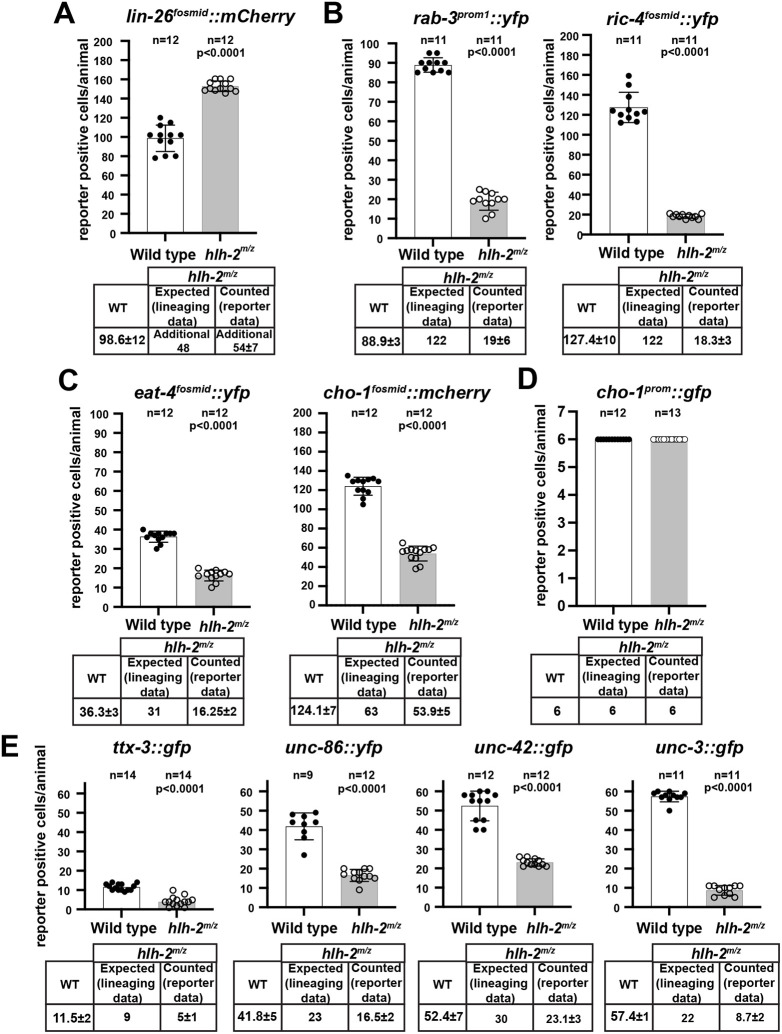
**Effect of loss of *hlh-2* on cell type-specific differentiation markers.** (A) Effect of *hlh-2^m/z^* removal on a hypodermal fate marker, *lin-26^fosmid^::mCherry* (*otIs466*). (B) Effect of *hlh-2^m/z^* removal on expression of pan-neuronal reporter *rab-3^prom1^::yfp* (*otIs287*) and *ric-4^fosmid^::yfp* (*otIs353*). (C) Effect of *hlh-2^m/z^* removal on glutamatergic and cholinergic neuron differentiation, as assessed by *eat-4^fosmid^::yfp* (*otIs388*) and *cho-1^fosmid^::mCherry* (*otIs544*). (D) Effect of *hlh-2^m/z^* removal on a differentiation marker specifically expressed in AIA, AIY and AIN neurons, a *cho-1^prom^::gfp* promoter fragment (*otIs379*). (E) Effect of *hlh-2^m/z^* removal on terminal selectors, as assessed with the reporter alleles *unc-42[ot986 (unc-42::gfp)]*, *unc-3[ot839(unc-3::gfp)]* and the fosmid reporters *wgIs68 (ttx-3^fosmid^::gfp)* and *otIs337* (*unc-86::yfp*). In all graphs, the sample size is indicated above each bar. Data is presented as mean±s.d.; statistical comparisons between wild-type and mutant (with *P*-value shown) were carried out using Welch's *t*-test.

We first set out to validate hypodermal transformations by examining the expression of a zinc-finger transcription factor, *lin-26*, a pan-hypodermal cell fate marker with instructive roles in hypodermal cell fate specification (Labouesse et al., 1994, 1996). Transgenic animals expressing a fosmid-based reporter for LIN-26 expression (*otIs466*) reveal expression in ∼50 additional cells in *hlh-2^m/z^* mutant embryos compared with wild-type embryos, thereby confirming the generation of additional hypodermal cells (Fig. 3A).

Mirroring our analysis of pan-hypodermal fate, we examined the expression of two pan-neuronal marker genes, *rab-3* (*RAB3*) and *ric-4* (*SNAP25*), encoding for proteins of the synaptic vesicle cycle (Stefanakis et al., 2015). In *hlh-2^m/z^* mutant embryos, we observed a striking reduction in the number of *rab-3::yfp(+)* and *ric-4::yfp(+)* cells, with fewer than 20 (of the 122 expected) cells expressing these two markers (Fig. 3B). On the one hand, this observation confirms the lineaging observation that neurons are still generated in *hlh-2^m/z^* mutant embryos and therefore that proneural bHLH genes are not responsible for all embryonic neurogenesis. On the other hand, the extent of *rab-3::yfp* and *ric-4::yfp* marker expression loss (<20 cells still express these markers) is much more expansive than expected from the lineage analysis, which predicted that 122 neurons appear to be generated normally by light microscopical lineage analysis.

As an alternative means to visualize neuronal identity acquisition, we assessed the expression of neurotransmitter identity features. To this end, we examined expression of fosmid-based reporters that measure the expression of the vesicular transporter *eat-4*, a marker of glutamatergic neuron differentiation, and *cho-1*, a general marker of cholinergic differentiation (Serrano-Saiz et al., 2013; Pereira et al., 2015). These two neurotransmitter systems cover the vast majority of neurons in the embryonically generated nervous system and are expressed in neurons that our lineage analysis suggests is unaffected in *hlh-2^m/z^* mutant animals, as well as in neurons that display lineage transformations (Table S2). We observed a ∼50% reduction in the total number of neurons expressing either *eat-4* or *cho-1* in *hlh-2^m/z^* mutant animals (Fig. 3C). As we still detect ∼16 *eat-4(+)* and ∼54 *cho-1(+)* neurons, we conclude that many neurons still execute at least a part of their differentiation program in *hlh-2^m/z^* mutant embryos. We determined the precise molecular identity of a subset of these unaffected neurons by examining the expression of an enhancer fragment derived from the *cho-1* choline transporter (Zhang et al., 2014). This fragment is expressed in six neurons (AIA, AIY and AIN neuron pairs) and is unaffected in *hlh-2^m/z^* mutant embryos, corroborating that these neurons differentiate normally in the absence of *hlh-2* (Fig. 3D).

The most striking aspect of our analysis of neurotransmitter identity markers is that the total number of *eat-4(+)* and *cho-1(+)* cells (70) far exceeds the number of cells that still express pan-neuronal markers (<20), indicating selective, identity feature-specific effects of *hlh-2* on neuron differentiation. We sought to further investigate this selectivity by turning to another set of marker genes, as described in the next section.

### Neuron type-specific effects of *hlh-2* on terminal selector expression

To further assess neuronal cell fate acquisition in *hlh-2^m/z^* mutant animals, we examined the expression of neuron type-specific terminal selector transcription factors (Hobert, 2008, 2016). These factors exert their activity shortly after neuron birth, initiating the expression of neuron type-specific gene batteries, but not pan-neuronal identity features (Hobert, 2016). Moreover, in a number of different lineages, terminal selectors have been found to be downstream of class II proneural bHLH genes that presumably work in conjunction with the class I heterodimerization partner HLH-2 (Masoudi et al., 2021). We analyzed the expression of four phylogenetically conserved terminal selectors, *ttx-3* (*LHX2/9*), *unc-86* (*POU4F1*), *unc-42* (*PROP1*) and *unc-3* (*EBF1/2/4*), which together mark the proper differentiation of about half of the 221 embryonically generated neurons (Finney and Ruvkun, 1990; Pereira et al., 2015; Reilly et al., 2020; Berghoff et al., 2021)*.*

The terminal selector *ttx-3*, a LIM homeobox gene, is continuously expressed in the AIY, AIN, AIA, NSM and ASK neuron pairs (Reilly et al., 2020), as well as transiently in the SMDD neuron pair (Bertrand and Hobert, 2009), a total of 12 neurons (Fig. 3E). As per our light microscopical Simi BioCell analysis, all these neurons appear to be normal in *hlh-2^m/z^* mutant embryos with the exception of the single ASKR neuron, for which a hypodermal cell fate transformation is observed. We found that in *hlh-2^m/z^* mutant embryos, *ttx-3* expression was reduced by about half, down to six cells (Fig. 3E). We reverse-lineaged some of the *ttx-3(+)* cells in *hlh-2^m/z^* mutant embryos and found that two of those remaining are the AIY interneuron pair (see Materials and Methods). We were surprised by this result because previous RNAi of *hlh-2* led to defects in *ttx-3* expression (Bertrand and Hobert, 2009; Filippopoulou et al., 2021), but we note that it is not unprecedented that RNAi-induced defects could not be reproduced with mutant alleles (Schmitz et al., 2007; Jimeno-Martín et al., 2022). As an independent means to assess AIY differentiation and effects on *ttx-3* expression, we examined the expression of a direct target gene of *ttx-3*, the choline transporter *cho-1* (Stefanakis et al., 2015). As noted above, expression of a *cho-1* enhancer fragment that is expressed in the AIY, and also AIN and AIA interneurons, is unaffected in *hlh-2^m/z^* mutant animals (Fig. 3D). We conclude from this analysis that, in some neurons where there appears to be no lineage defects, *hlh-2* indeed has no effect on differentiation (AIY, AIA, AIN), but that in other neurons (likely the remaining NSM, ASK, SMDD neurons), *hlh-2* affects neuronal differentiation based on the reduction of *ttx-3(+)* neurons.

A similar picture emerges when considering expression of the *unc-86* POU homeobox gene – another terminal selector that acts in multiple neuron types, in combination with other homeobox genes, to specify their identity (Leyva-Díaz et al., 2020). In the embryo, *unc-86* has been reported to be selectively expressed in 46 neurons (Finney and Ruvkun, 1990), a number we confirmed with our reporter transgene (Fig. 3E). Lineage analysis predicts a transformation of 23 of the neurons to hypodermal fate or programmed cell death, with the other 23 neurons appearing to be generated normally, as assessed by their neuronal nuclear morphology (Fig. 2; Table S3). We observe ∼16 neurons to express *unc-86* in *hlh-2^m/z^* mutant embryos. As with *ttx-3*, this again indicates that many cells indeed differentiate appropriately to express *unc-86*, but because only 16 out of an expected 23 cells do so, we conclude that a subset of those neuron-like cells fail to differentiate properly (Fig. 3E).

The *unc-42* homeobox gene is selectively expressed in 42 neurons during larval and adult stages (Berghoff et al., 2021) and our lineaging shows 12 of these neurons to be transformed to hypodermal fate or programmed cell death (Fig. 2; Table S3). Analysis of an *unc-42* reporter allele (*ot986*) reveals more expression in wild-type embryos (>50 cells), indicating previously undescribed transient *unc-42* expression in additional cells. Almost half these cells lose *unc-42* expression in *hlh-2^m/z^* mutant embryos (Fig. 3E). As it is not clear whether this reduction is due to loss of expression in these transiently UNC-42(+) neurons, we reverse-lineaged cells and found that UNC-42(+) cells in *hlh-2^m/z^* mutant embryos include ASH, AVK, SIB and SMD [12 of the 23 UNC-42(+) neurons]. These neurons do not show lineage transformation in our embryonic lineaging experiments and we therefore again conclude that several neuronal cell types acquire their neuron-specific identities in *hlh-2^m/z^* mutant embryos, whereas others do not. We further confirmed this notion by using another marker for terminal differentiation of AVK, the FMRF-amide encoding *flp-1* gene, a target of UNC-42 (Wightman et al., 2005). We found *flp-1::gfp* (*bwIs2* transgene) expression to be unaffected in 15/15 examined *hlh-2^m/z^* mutants.

Lastly, we examined expression of *unc-3*, the sole *C. elegans* ortholog of Collier/Olf/Ebf, which acts as terminal selector in several cholinergic motor neuron types (Kratsios et al., 2011; Pereira et al., 2015). As in the case of *unc-42*, we observed more widespread embryonic expression of *unc-3* in wild-type embryos (∼57 cells) than predicted by previous expression pattern analysis in the postembryonic nervous system (34 cells). Of the 34 neurons that express *unc-3* postembryonically, 22 are supposedly normally generated in *hlh-2^m/z^* mutants based on our lineage analysis (Fig. 2; Table S2). However, we observed an extensive reduction of *unc-3* reporter allele expression, with only around nine UNC-3(+) cells observed in *hlh-2^m/z^* mutant embryos (Fig. 3E; Table S2). This again indicates that many neuron-like cells (as indicated by lineage analysis) fail to differentiate properly in *hlh-2^m/z^* mutant embryos, while other neuron-like cells do differentiate properly.

## DISCUSSION

**Fig. 4.**
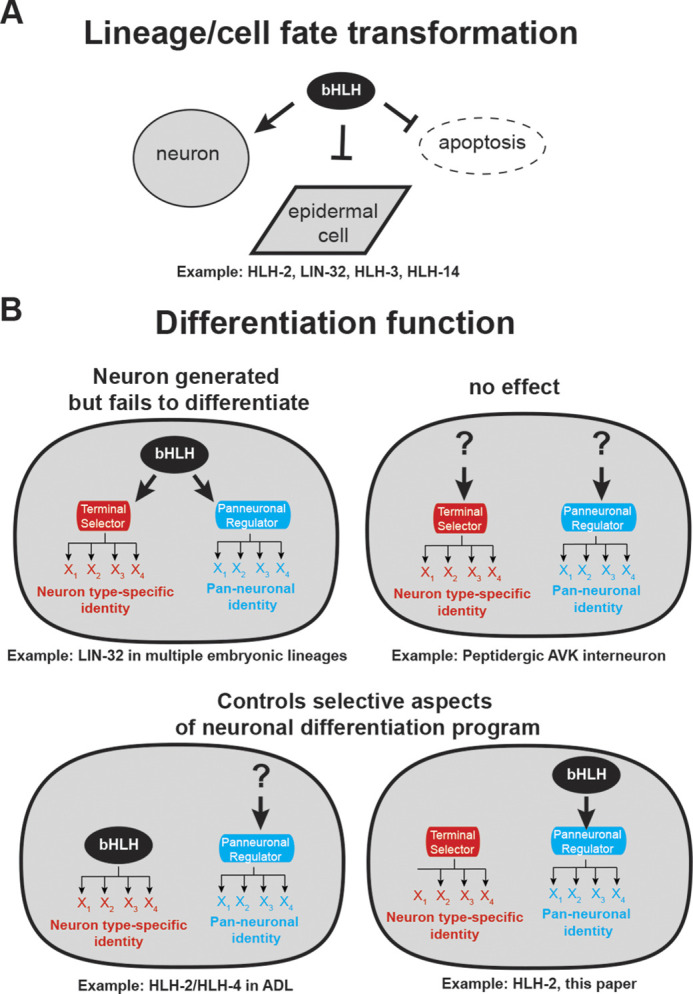
**Summary of involvement of the ‘proneural’ bHLH gene family (class I *hlh-2* and class II AS-C-like and Ato-like genes) in neuronal identity specification.** (A) Loss of class I (HLH-2) and several class II bHLH protein results in, ‘classic’ proneural phenotypes, manifested by a transformation to hypo/epidermal cell fate (Zhao and Emmons, 1995; Portman and Emmons, 2000; Frank et al., 2003; Nakano et al., 2010; Poole et al., 2011; Luo and Horvitz, 2017; Masoudi et al., 2021), analogous to proneural defects originally described in the *Drosophila* nervous system (Campuzano and Modolell, 1992; Jarman et al., 1993). (B) Consequences of loss of class I and class II ‘proneural’ bHLH genes on neuronal differentiation in *C. elegans.* Neuronal differentiation is broken down here in genetically separable processes: the control neuron type-specific gene batteries (via terminal selectors) and pan-neuronal gene batteries (via the CUT homeobox genes) (Leyva-Diaz and Hobert, 2022). See text for more details.

Extensive work on neuronal cell fate specification in *C. elegans* has revealed some themes that are broadly applicable throughout the entire *C. elegans* nervous system. These include coordinated regulation of neuron type-specific terminal identity features by so-called terminal selectors, the separate regulation of neuron type-specific terminal identity features from pan-neuronal gene regulation and the deployment of homeobox genes as neuronal identity specifiers throughout the entire nervous system of the worm (Hobert, 2016, 2021). The existence of such universal themes prompted us to ask whether all *C. elegans* neurons also rely on a common mechanism for earlier steps of neuronal development: specifically, the decision to generate neuronal precursors from ectodermal-derived cells. In several different cellular contexts, so-called ‘proneural bHLH genes’ (AS-C and Ath family members), were already known to fulfill such roles in many different animal species (Jan and Jan, 1994; Hassan and Bellen, 2000; Bertrand et al., 2002; Wang and Baker, 2015; Baker and Brown, 2018), yet a true nervous system-wide perspective of the involvement of these proneural bHLH genes in neurogenesis was lacking. Through the removal of the common proneural complex subunit HLH-2, we have addressed here the question of whether proneural bHLH genes can be made responsible for all neurogenesis in the *C. elegans* embryo. We draw the following three conclusions from our analysis (Fig. 4).

First, as expected, *hlh-2* – likely in combination with proneural class II bHLH genes – does have a proneural function in many lineages throughout the central and peripheral nervous system of *C. elegans* with no particular preference for lineage, neuron position or neuron function*.* The most obvious manifestation of such a role is the transformation of neuronal lineages or cells to hypodermal cell fates, a ‘classic’ phenotype of proneural genes, initially observed in the peripheral nervous system of *Drosophila* (Jan and Jan, 1994; Campuzano and Modolell, 1992; Jaman et al., 1993) and subsequently in *C. elegans* (Zhao and Emmons, 1995) (Fig. 4). Such hypodermal transformations are only the most extreme version of a proneural function. Based on our previous analysis of *lin-32* function (Masoudi et al., 2021), and confirmed here with our analysis of *hlh-2*, proneural functions are also manifested by a combined loss of terminal selectors, pan-neuronal markers and neuron type-specific markers without concomitant switch to hypodermal identity (Fig. 4B, upper right panel).

Second, based on our lineage and marker gene analysis, we conclude that not all neurogenic processes in an animal nervous system require canonical class I/II proneural bHLH complexes. This is a novel conclusion that could not be made in any other system before and is based on our ability to analyze neurogenesis throughout an entire nervous system. Neurons that are affected or appear to be left unaffected by removal of *hlh-2* do not share any specific common themes in terms of overall location, function or lineage history. One potential caveat of our analysis is that we cannot be certain that the *hlh-2* allele that we used is a molecular null allele. Arguing against this possibility, animals that carry the allele that we used for our analysis, a splice site mutation, show similar embryonic arrest phenotypes as an *hlh-2* deletion allele (Nakano et al., 2010). However, we could not test the deletion allele for neurogenic defects because of our inability to balance this allele with a rescuing array (see Materials and Methods).

One may not have to look far to find alternative proneural factors. A recent report indicates that the two conventional proneural AS-C/ATO-like genes (*hlh-3* and *ngn-1*) act synergistically with a bHLH gene not previously considered to be a proneural factor, the OLIG-homolog *hlh-16*, to affect the proper differentiation of the AIY interneuron class, as measured by expression of the terminal selector *ttx-3* (Filippopoulou et al., 2021). We found AIY to be unaffected by *hlh-2* removal*.* Both the redundant function of these factors (neither single mutant alone has a phenotype) (Filippopoulou et al., 2021), as well as their apparent independence of *hlh-2*, suggests proneural function of non-canonical class II-only bHLH complexes.

Third, there are striking discrepancies in the extent by which distinct types of neuronal marker genes are affected in *hlh-2* mutants, indicating that *hlh-2* has nuanced, cell-type functions in controlling select aspects of terminal neuron differentiation (Fig. 4B, lower panels). Specifically, the effects of *hlh-2* removal on pan-neuronal genes appear to be broader than expected from the expression of neuron type-specific identity markers (i.e. terminal selectors and other neuron type-specific effector genes). In other words, in several lineages, *hlh-2* may control pan-neuronal features independently of neuron type-specific differentiation programs. One limitation of our study is that we cannot pinpoint the nature of these neurons due to the overall morphological disorganization of *hlh-2^m/z^* mutant embryos. This conclusion therefore relies only on counting the number of fate marker-positive cells (e.g. pan-neuronal marker genes versus neuron type-specific marker genes) and comparing them with each other in wild-type and *hlh-2* mutant animals. In spite of this limitation, we note that this conclusion is consistent with previous reports that showed that pan-neuronal differentiation can be genetically separated from neuron type-specific differentiation. Specifically, terminal selectors affect neuron type-specific features, but leave pan-neuronal differentiation programs unaffected; conversely, members of the CUT homeobox gene family directly activate the expression of pan-neuronal identity features but leave neuron-specific identity features intact (Hobert, 2016; Leyva-Díaz and Hobert, 2022). Hence, it is conceivable that HLH-2 complexes may either directly control pan-neuronal effector genes and/or may control the expression of CUT homeobox genes; however, this cannot be a universal function, as pan-neuronal features remain unaffected in several neuronal lineages.

In summary, nervous system-wide analysis of class I and class II bHLH gene function, reported here and in recent papers from our laboratory (Masoudi et al., 2018, 2021), provides novel and more nuanced views of proneural gene function in the nervous system of *C. elegans* that we summarize in Fig. 4.

## MATERIALS AND METHODS

### Mutant strains

*Caenorhabditis elegans* strains were maintained by standard methods (Brenner, 1974). Mutant strains: MT17677 – *hlh-2(n5053) / hT2[qIs48] I;+/ hT2[qIs48] III* (Nakano et al., 2010); MT19085 – *hlh-2(n5287) / hT2[qIs48] I;+/ hT2[qIs48] III* (Nakano et al., 2010); OH16873 – *hlh-2(n5053); otEx7684* (*hlh-2^fosmid^::yfp; myo-3::mcherry*) (this paper).

### Reporter alleles and transgenes

CRISPR/Cas9-engineered reporter alleles: OH16761 – *hlh-2[ot1089 (hlh-2::gfp)]* (this paper); OH16111 – *unc-42[ot986 (unc-42::gfp)]* (Berghoff et al., 2021); OH13990 – *unc-3[ot839(unc-3::gfp)]* (Kratsios et al., 2017).

Reporter transgenes: *otIs502* – *hlh-2^fosmid^::yfp+myo-3p::mCherry* (Sallee et al., 2017); *otIs466* – *lin-26^fosmid^::mCherry; lin-44::nls::yfp* (this paper); *otIs353* – *ric-4^fosmid^::yfp* (Stefanakis et al., 2015); *otIs287* – *rab-3^prom1^::yfp* (Stefanakis et al., 2015); *otIs388* – *eat-4^fosmid^::yfp::H2B* (Serrano-Saiz et al., 2013); *otIs544* – *cho-1^fosmid^::SL2::NLS::mCherry* (Pereira et al., 2015); *otIs379* – *cho-1^prom^::gfp* (Zhang et al., 2014); *bwIs2* – *flp-1::gfp* (Much et al., 2000); *otIs337* – *unc-86^fosmid^::yfp* (Pereira et al., 2015); *wgIs68* – *ttx-3^fosmid^::gfp* (Zhang et al., 2014).

We also examined expression of the two remaining AS-C homologs, *hlh-6* and *hlh-19*, and found neither to be expressed in the nervous system. A 5′ promoter fusion reporter upstream of the *hlh-6* locus is exclusively expressed in pharyngeal gland cells (Smit et al., 2008). To exclude the possibility that regulatory elements were missed in this reporter, we generated a fosmid-based reporter of *hlh-6* using fosmid recombineering (Tursun et al., 2009) and also found it to be exclusively expressed in pharyngeal gland cells. We also generated a fosmid-based reporter for the previously unstudied *hlh-19* gene and found it to be weakly expressed in embryonic hypodermal cells but not in the developing nervous system. Tagging the endogenous *hlh-19* locus with *gfp* using CRISPR/Cas9 genome engineering, carried out by SunyBiotech, revealed no expression in any tissue.

### Maternal/zygotic *hlh-2* removal

The first indication that zygotic *hlh-2* mutants may not fully remove all gene activity was the variability in expressivity of arrest phenotypes that homozygous *hlh-2(n5053)* mutants, derived from heterozygous mothers, displayed (Nakano et al., 2010). Animals arrested at different embryonic stages and some animals even survived till the first larval stage. To remove potential maternal contribution, we rescued the embryonic lethality of homozygous *n5053* mutant animals with a fosmid (WRM0610dG01) encompassing the entire *hlh-2* locus, tagged with *gfp* by fosmid recombineering (Fig. 1) (Tursun et al., 2009). A transgenic array (*otEx7684*) expressing this fosmid shows, like all transgenic array, an inherent mitotic instability. Via a co-injection marker expressed in body wall muscle (*myo-3::mCherry*), animals can be identified that have lost the *hlh-2* rescuing array in the P lineage (generating muscle and germline) of the parental generation and hence contain neither maternal nor zygotic gene activity. The effect of maternal contribution was most obvious when examining expression of the pan-neuronal *rab-3* marker. Although zygotic mutants showed no loss of *rab-3* expression, *n5053* homozygous animals derived from mother that did not contain the rescuing array in the germline forming P2 lineage (*hlh-2^m/z^* mutants) showed a reduced number of *rab-3(+)* cells, demonstrating the impact of maternally supplied *hlh-2* on neurogenesis.

In spite of repeated attempts with multiple different genomic fosmid clones, we were not able to rescue the embryonic lethality of the *n5287* deletion allele. When crossed into *n5287* animals, even the transgenic array that rescued the *n5053* allele (*otEx7689*) could not rescue the *n5287* lethality. We therefore cannot assign conclusively the embryonic lethality of *n5287* animals exclusively to the *hlh-2* locus and therefore used the *n5053* allele for all of our analysis.

### Microscopy and lineaging

For fluorescence microscopy, worms were paralyzed by 25 mM sodium azide (NaN_3_) and mounted on a 5% agarose pad on glass slides. Images were acquired using an axioscope (Zeiss, AXIO Imager Z.2) or LSM 800 laser point scanning confocal microscope (Zeiss). Representative images are maximum-projection of *z*-stacks. Image reconstruction was performed using Fiji software (Schindelin et al., 2012).

Simi BioCell and 4D microscopy was used, as previously described (Schnabel et al., 1997), to analyze embryonic lineage defects of mutant animals as well as reporter expression pattern during embryogenesis. Briefly, gravid adults were dissected on glass slides and a single two-cell stage embryo was mounted and recorded over 8 h of embryonic development. Nomarski stacks were taken every 30 s and embryos were illuminated with LED fluorescence light (470 nm) at set time points during development. The recording was carried out using a Zeiss Imager Z1 compound microscope, using the 4D microscopy software Steuerprogram (Caenotec).

We conducted ‘reverse lineaging’ experiment to determine the identity of *ttx-3::gfp*- and *unc-42::gfp*-expressing neurons. Similar to normal lineaging, *hlh-2^m/z^* mutant embryos were mounted at the two-cell stage and embryonic development was recorded for almost 8 h. The resulting video was analyzed using Simi BioCell. Compared with forward lineaging, in which we trace each cell by moving forward in time, starting from a 20-cell staged embryo toward a 1.5-fold embryo, in reverse lineaging we moved back in time starting from 1.5-fold embryo towards a two-cell stage embryo. To this end, any cell expressing the reporter of choice at the 1.5-fold stage was selected blindly, not knowing its identity, and lineaged backward toward the beginning of recording when the embryo was two-cell staged. Once this lineage was completed, it was mapped on the canonical lineage map. Based on the position of ancestral cells and the pattern of division we can confidently identify the cell that was expressing the reporter of choice. Our reverse lineaging also showed that there are no delays of onset of *ttx-3::gfp* and *unc-42::gfp* expression in *hlh-2^m/z^* mutant animals.

## Supplementary Material

10.1242/develop.201366_sup1 Supplementary information
