# Supplementary material for: Cell-specific effects of the sole C. elegans Daughterless/E protein homolog, HLH-2, on nervous system development
Source: Development. 2023 Jan 3;150(1):dev201366. doi: 10.1242/dev.201366 (PMC10108603; doi:10.1242/dev.201366)
Supplement: Supplementary information [file develop-150-201366-s1.pdf]

**Table S1. Tabular comparison of all proneural bHLH expression profile**

A summary of the complete developmental expression patterns of the sole proneural Atonal ortholog *lin-32*, the sole NeuroD ortholog *cnd-1*, the sole neurogenin ortholog *ngn-1*, and three of the five AS-C homologs (*hlh-3, hlh-4, hlh-14*) has been generated using fosmid-based reporter transgenes and/or CRISPR/Cas9-engineered reporter alleles (Masoudi et al. 2018; Masoudi et al. 2021). Colored box indicates that the respective gene is expressed at any point in the lineage that generates the indicated neuron type.

| Class | Subclass | *hlh-2* | *hlh-3* | *hlh-14* | *cnd-1* | *ngn-1* | *lin-32* |
|---|---|---|---|---|---|---|---|
| ADA | L | ■ | ■ | | | | ■ |
| | R | ■ | ■ | | | | ■ |
| ADE | L | ■ | ■ | | | | ■ |
| | R | ■ | ■ | | | | ■ |
| ADF | L | ■ | ■ | ■ | | | |
| | R | ■ | ■ | ■ | | | |
| ADL | L | ■ | ■ | ■ | | | |
| | R | ■ | ■ | ■ | | | |
| AFD | L | ■ | ■ | ■ | | | |
| | R | ■ | ■ | ■ | | | |
| AIA | L | ■ | ■ | | | ■ | |
| | R | ■ | ■ | | | ■ | |
| AIB | L | ■ | ■ | ■ | | | ■ |
| | R | ■ | ■ | | | | |
| AIM | L | ■ | ■ | | ■ | | ■ |
| | R | ■ | ■ | | ■ | | ■ |
| AIN | L | ■ | ■ | | | ■ | |
| | R | ■ | ■ | | | ■ | |
| AIY | L | ■ | ■ | | | | |
| | R | ■ | ■ | ■ | | ■ | |
| AIZ | L | ■ | ■ | | | | ■ |
| | R | ■ | ■ | | | | ■ |
| ALA | | ■ | ■ | ■ | | ■ | |
| ALM | L | ■ | ■ | ■ | | | ■ |
| | R | ■ | ■ | ■ | | | ■ |
| ALN | L | ■ | ■ | | | | ■ |
| | R | ■ | ■ | | | | ■ |
| ASE | L | ■ | ■ | ■ | | | |
| | R | ■ | ■ | ■ | ■ | | |
| ASG | L | ■ | ■ | ■ | | | |
| | R | ■ | ■ | | | | |
| ASH | L | ■ | ■ | | ■ | | |
| | R | ■ | ■ | | ■ | | |
| ASI | L | ■ | ■ | ■ | | | ■ |
| | R | ■ | ■ | | | | |
| ASJ | L | ■ | ■ | ■ | | | |
| | R | ■ | ■ | ■ | ■ | | |
| ASK | L | ■ | ■ | ■ | | | ■ |
| | R | ■ | ■ | ■ | | | ■ |
| AUA | L | ■ | ■ | ■ | | | |
| | R | ■ | ■ | ■ | ■ | | |
| AVA | L | ■ | ■ | | | | |
| | R | ■ | ■ | | | ■ | |
| AVB | L | ■ | ■ | | ■ | | |
| | R | ■ | ■ | | ■ | ■ | |
| AVD | L | ■ | ■ | | | ■ | |
| | R | ■ | ■ | | | ■ | |
| AVE | L | ■ | ■ | ■ | | | |
| | R | ■ | ■ | ■ | | | |
| AVG | | ■ | ■ | ■ | | | |
| AVH | L | ■ | ■ | | | ■ | ■ |
| | R | ■ | ■ | ■ | | ■ | ■ |
| AVJ | L | ■ | | | | ■ | ■ |
| | R | ■ | ■ | ■ | | ■ | |
| AVK | L | ■ | ■ | | | | |
| | R | ■ | ■ | ■ | | | |
| AVL | | ■ | ■ | ■ | | | |
| AWA | L | ■ | ■ | ■ | | | |
| | R | ■ | ■ | | | | |
| AWB | L | ■ | ■ | ■ | | | |
| | R | ■ | ■ | ■ | | | |
| AWC | L | ■ | ■ | | ■ | ■ | |
| | R | ■ | ■ | | ■ | ■ | |
| BAG | L | ■ | ■ | | ■ | | |
| | R | ■ | ■ | | ■ | | |
| BDU | L | ■ | ■ | ■ | | | ■ |
| | R | ■ | ■ | ■ | | | ■ |
| CAN | L | ■ | ■ | | | ■ | ■ |
| | R | ■ | ■ | ■ | | ■ | |
| CEP | DL | ■ | ■ | | | | ■ |
| | DR | ■ | ■ | | | | ■ |
| | VL | ■ | ■ | | ■ | | ■ |
| | VR | ■ | ■ | | ■ | | ■ |

| Class | Subclass | *hlh-2* | *hlh-3* | *hlh-14* | *cnd-1* | *ngn-1* | *lin-32* |
|---|---|---|---|---|---|---|---|
| DA | 1 | ■ | ■ | ■ | | ■ | |
| | 2 | ■ | ■ | | ■ | ■ | |
| | 3 | ■ | ■ | ■ | | ■ | |
| | 4 | ■ | ■ | | ■ | ■ | |
| | 5 | ■ | ■ | ■ | | ■ | |
| | 6 | ■ | ■ | ■ | | ■ | |
| | 7 | ■ | ■ | ■ | | ■ | |
| | 8 | ■ | ■ | ■ | | ■ | |
| | 9 | ■ | ■ | ■ | | ■ | |
| DB | 1 | ■ | ■ | | ■ | | |
| | 2 | ■ | ■ | | ■ | ■ | |
| | 3 | ■ | ■ | | ■ | | |
| | 4 | ■ | ■ | ■ | | | |
| | 5 | ■ | ■ | | | | |
| | 6 | ■ | ■ | | | ■ | |
| | 7 | ■ | ■ | | | ■ | |
| DD | 1 | ■ | ■ | | ■ | | |
| | 2 | ■ | ■ | ■ | | ■ | |
| | 3 | ■ | ■ | | ■ | | |
| | 4 | ■ | ■ | ■ | | ■ | |
| | 5 | ■ | ■ | | ■ | | |
| | 6 | ■ | ■ | ■ | | ■ | |
| DVA | | ■ | ■ | ■ | | | |
| DVC | | ■ | ■ | ■ | | | |
| FLP | L | ■ | ■ | | | | ■ |
| | R | ■ | ■ | | | | ■ |
| HSN | L | ■ | ■ | ■ | | | ■ |
| | R | ■ | ■ | ■ | | | ■ |
| I1 | L | ■ | ■ | | | | |
| | R | | ■ | | | ■ | |
| I2 | L | ■ | ■ | | | | |
| | R | | ■ | | | ■ | |
| I3 | | ■ | ■ | | | ■ | |
| I4 | | ■ | ■ | | | ■ | |
| I5 | | | ■ | | | ■ | |
| I6 | | ■ | ■ | | | ■ | |
| IL1 | DL | ■ | | | | ■ | ■ |
| | DR | ■ | ■ | ■ | | ■ | ■ |
| | L | ■ | ■ | ■ | | ■ | ■ |
| | R | ■ | ■ | ■ | | ■ | ■ |
| | VL | ■ | ■ | | ■ | | ■ |
| | VR | ■ | ■ | | ■ | ■ | ■ |
| IL2 | DL | ■ | | | | ■ | ■ |
| | DR | ■ | ■ | ■ | | ■ | ■ |
| | L | ■ | ■ | ■ | | ■ | ■ |
| | R | ■ | ■ | ■ | | ■ | ■ |
| | VL | ■ | ■ | | ■ | | ■ |
| | VR | ■ | ■ | | ■ | ■ | ■ |
| LUA | L | ■ | ■ | ■ | | ■ | |
| | R | ■ | ■ | ■ | | ■ | |
| M1 | | ■ | ■ | | | ■ | |
| M2 | L | ■ | ■ | | | ■ | |
| | R | ■ | ■ | ■ | | ■ | |
| M3 | L | ■ | ■ | | | ■ | |
| | R | ■ | ■ | ■ | | ■ | |
| M4 | | ■ | ■ | | | ■ | |
| M5 | | ■ | ■ | | | ■ | |
| MC | L | ■ | ■ | | | ■ | |
| | R | | ■ | | | | |
| MI | | ■ | ■ | | | ■ | |
| NSM | L | | ■ | | | ■ | |
| | R | | ■ | ■ | | ■ | |
| OLL | L | ■ | ■ | ■ | | | ■ |
| | R | ■ | ■ | ■ | | | ■ |
| OLQ | DL | ■ | | | | ■ | ■ |
| | DR | ■ | ■ | ■ | | ■ | ■ |
| | VL | ■ | ■ | | ■ | | ■ |
| | VR | ■ | ■ | | ■ | | ■ |
| PHA | L | ■ | ■ | ■ | | ■ | |
| | R | ■ | ■ | ■ | | ■ | |
| PHB | L | ■ | ■ | ■ | | | ■ |
| | R | ■ | ■ | ■ | | | ■ |
| PLM | L | ■ | ■ | | | | ■ |
| | R | ■ | ■ | | | | ■ |

| Class | Subclass | *hlh-2* | *hlh-3* | *hlh-14* | *cnd-1* | *ngn-1* | *lin-32* |
|---|---|---|---|---|---|---|---|
| PVC | L | ■ | ■ | ■ | | ■ | |
| | R | ■ | ■ | ■ | | ■ | |
| PVP | L | ■ | ■ | ■ | | | |
| | R | ■ | ■ | ■ | | | |
| PVQ | L | ■ | ■ | ■ | | | |
| | R | ■ | ■ | ■ | | | |
| PVT | | ■ | ■ | | | | |
| RIA | L | ■ | ■ | ■ | | ■ | ■ |
| | R | ■ | ■ | ■ | | ■ | |
| RIB | L | ■ | ■ | | ■ | | |
| | R | ■ | ■ | | ■ | | |
| RIC | L | ■ | ■ | | | ■ | |
| | R | ■ | ■ | | | ■ | |
| RID | | ■ | ■ | ■ | | ■ | |
| RIF | L | ■ | ■ | | ■ | ■ | |
| | R | ■ | ■ | ■ | | ■ | |
| RIG | L | ■ | ■ | | ■ | | |
| | R | ■ | ■ | ■ | | ■ | |
| RIH | | ■ | ■ | | | | |
| RIM | L | ■ | ■ | | | ■ | |
| | R | ■ | ■ | | | ■ | |
| RIP | L | ■ | ■ | | ■ | | |
| | R | ■ | ■ | | ■ | ■ | |
| RIR | | ■ | ■ | ■ | | | |
| RIS | | ■ | ■ | ■ | | | |
| RIV | L | ■ | ■ | | ■ | | |
| | R | ■ | ■ | | ■ | ■ | |
| RMD | DL | ■ | ■ | | ■ | | |
| | DR | ■ | ■ | | ■ | ■ | |
| | L | ■ | ■ | ■ | | | |
| | R | ■ | ■ | ■ | | | |
| | VL | ■ | ■ | | ■ | | |
| | VR | ■ | ■ | | ■ | ■ | |
| RME | DL | ■ | ■ | ■ | | ■ | |
| | DR | ■ | | | ■ | ■ | |
| | VL | ■ | | | ■ | ■ | |
| | VR | ■ | ■ | | | | |
| RMG | L | ■ | ■ | | | | ■ |
| | R | ■ | ■ | | | | ■ |
| SAA | DL | ■ | ■ | | ■ | | |
| | DR | ■ | ■ | | ■ | ■ | |
| | VL | ■ | ■ | | ■ | ■ | |
| | VR | ■ | ■ | | ■ | ■ | |
| SAB | D | ■ | ■ | | ■ | ■ | |
| | VL | ■ | ■ | | ■ | ■ | |
| | VR | ■ | ■ | ■ | | ■ | |
| SIA | DL | ■ | ■ | | | | |
| | DR | ■ | ■ | ■ | | ■ | |
| | VL | ■ | ■ | | | | |
| | VR | ■ | ■ | ■ | | ■ | |
| SIB | DL | ■ | ■ | | | ■ | |
| | DR | ■ | ■ | | | ■ | |
| | VL | ■ | ■ | | | | |
| | VR | ■ | ■ | ■ | | ■ | |
| SMB | DL | ■ | ■ | | ■ | | |
| | DR | ■ | ■ | | ■ | ■ | |
| | VL | ■ | ■ | | ■ | | |
| | VR | ■ | ■ | | ■ | ■ | |
| SMD | DL | ■ | ■ | | | | |
| | DR | ■ | ■ | ■ | | ■ | |
| | VL | ■ | ■ | | ■ | ■ | |
| | VR | ■ | ■ | | ■ | ■ | |
| URA | DL | ■ | ■ | | | | ■ |
| | DR | ■ | ■ | | | | ■ |
| | VL | ■ | ■ | | ■ | | |
| | VR | ■ | ■ | | ■ | | |
| URB | L | ■ | ■ | ■ | | | |
| | R | ■ | ■ | | | | |
| URX | L | ■ | ■ | | | | ■ |
| | R | ■ | ■ | | | | ■ |
| URY | DL | ■ | | | | ■ | ■ |
| | DR | ■ | ■ | ■ | | ■ | ■ |
| | VL | ■ | ■ | | ■ | | ■ |
| | VR | ■ | ■ | | ■ | | ■ |

Development • Supplementary information

**Table S2. Tabular summary of neuron cell fate loss in *hlh-2*$^{m/z}$ mutant animals**. All embryonically generated neurons, their examined neurotransmitter identities and resident terminal selectors are shown (their expression is analyzed in **Fig.3**). Color coding indicates transformation to cell death and to hypodermal fate. Also see **Table S3** for a summary of cell fate transformation, organized by neuronal cell type.

| Class | Neuron | Transform in *hlh-2(-)* | NT identity | Terminal selector |
|---|---|---|---|---|
| ADA | ADAL | | | |
| | ADAR | | | |
| ADE | ADEL | | | |
| | ADER | | | |
| ADF | ADFL | | | |
| | ADFR | | | |
| ADL | ADLL | | | |
| | ADLR | | | |
| AFD | AFDL | | | |
| | AFDR | | | |
| AIA | AIAL | | | |
| | AIAR | | | |
| AIB | AIBL | | | |
| | AIBR | | | |
| AIM | AIML | | | |
| | AIMR | | | |
| AIN | AINL | | | |
| | AINR | | | |
| AIY | AIYL | | | |
| | AIYR | | | |
| AIZ | AIZL | | | |
| | AIZR | | | |
| ALA | ALA | | | |
| ALM | ALML | | | |
| | ALMR | | | |
| ALN | ALNL | | | |
| | ALNR | | | |
| ASE | ASEL | | | |
| | ASER | | | |
| ASG | ASGL | | | |
| | ASGR | | | |
| ASH | ASHL | | | |
| | ASHR | | | |
| ASI | ASIL | | | |
| | ASIR | | | |
| ASJ | ASJL | | | |
| | ASJR | | | |
| ASK | ASKL | | | |
| | ASKR | | | |
| AUA | AUAL | | | |
| | AUAR | | | |
| AVA | AVAL | | | |
| | AVAR | | | |
| AVB | AVBL | | | |
| | AVBR | | | |
| AVD | AVDL | | | |
| | AVDR | | | |
| AVE | AVEL | | | |
| | AVER | | | |
| AVG | AVG | | | |
| AVH | AVHL | | | |
| | AVHR | | | |
| AVJ | AVJL | | | |
| | AVJR | | | |
| AVK | AVKL | | | |
| | AVKR | | | |
| AVL | AVL | | | |
| AWA | AWAL | | | |
| | AWAR | | | |
| AWB | AWBL | | | |
| | AWBR | | | |
| AWC | AWCL | | | |
| | AWCR | | | |
| BAG | BAGL | | | |
| | BAGR | | | |
| BDU | BDUL | | | |
| | BDUR | | | |
| CAN | CANL | | | |
| | CANR | | | |
| CEP | CEPDL | | | |
| | CEPDR | | | |
| | CEPVL | | | |
| | CEPVR | | | |
| DA | DA1 | | | |
| | DA2 | | | |
| | DA3 | | | |
| | DA4 | | | |
| | DA5 | | | |
| | DA6 | | | |
| | DA7 | | | |
| | DA8 | | | |
| | DA9 | | | |
| DB | **DB1**/3 | | | |
| | DB2 | | | |
| | **DB3**/1 | | | |
| | DB4 | | | |
| | DB5 | | | |
| | DB6 | | | |
| | DB7 | | | |
| DD | DD1 | | | |
| | DD2 | | | |
| | DD3 | | | |
| | DD4 | | | |
| | DD5 | | | |
| | DD6 | | | |
| DVA | DVA | | | |
| DVC | DVC | | | |
| FLP | FLPL | | | |
| | FLPR | | | |
| HSN | HSNL | | | |
| | HSNR | | | |
| I1 | I1L | | | |
| | I1R | | | |
| I2 | I2L | | | |
| | I2R | | | |
| I3 | I3 | | | |
| I4 | I4 | | | |
| I5 | I5 | | | |
| I6 | I6 | | | |
| IL1 | IL1DL | | | |
| | IL1DR | | | |
| | IL1L | | | |
| | IL1R | | | |
| | IL1VL | | | |
| | IL1VR | | | |
| IL2 | IL2DL | | | |
| | IL2DR | | | |
| | IL2L | | | |
| | IL2R | | | |
| | IL2VL | | | |
| | IL2VR | | | |

| Class | Neuron | Transform in *hlh-2(-)* | NT identity | Terminal selector |
|---|---|---|---|---|
| LUA | LUAL | | | |
| | LUAR | | | |
| M1 | M1 | | | |
| M2 | M2L | | | |
| | M2R | | | |
| M3 | M3L | | | |
| | M3R | | | |
| M4 | M4 | | | |
| M5 | M5 | | | |
| MC | MCL | | | |
| | MCR | | | |
| MI | MI | | | |
| NSM | NSML | | | |
| | NSMR | | | |
| OLL | OLLL | | | |
| | OLLR | | | |
| OLQ | OLQDL | | | |
| | OLQDR | | | |
| | OLQVL | | | |
| | OLQVR | | | |
| PHA | PHAL | | | |
| | PHAR | | | |
| PHB | PHBL | | | |
| | PHBR | | | |
| PLM | PLML | | | |
| | PLMR | | | |
| PVC | PVCL | | | |
| | PVCR | | | |
| PVP | PVPL | | | |
| | PVPR | | | |
| PVQ | PVQL | | | |
| | PVQR | | | |
| PVR | PVR | | | |
| PVT | PVT | | | |
| RIA | RIAL | | | |
| | RIAR | | | |
| RIB | RIBL | | | |
| | RIBR | | | |
| RIC | RICL | | | |
| | RICR | | | |
| RID | RID | | | |
| RIF | RIFL | | | |
| | RIFR | | | |
| RIG | RIGL | | | |
| | RIGR | | | |
| RIH | RIH | | | |
| RIM | RIML | | | |
| | RIMR | | | |
| RIP | RIPL | | | |
| | RIPR | | | |
| RIR | RIR | | | |
| RIS | RIS | | | |
| RIV | RIVL | | | |
| | RIVR | | | |
| RMD | RMDDL | | | |
| | RMDDR | | | |
| | RMDL | | | |
| | RMDR | | | |
| | RMDVL | | | |
| | RMDVR | | | |
| RME | RMED | | | |
| | RMEL | | | |
| | RMER | | | |
| | RMEV | | | |
| RMG | RMGL | | | |
| | RMGR | | | |
| SAA | SAADL | | | |
| | SAADR | | | |
| | SAAVL | | | |
| | SAAVR | | | |
| SAB | SABD | | | |
| | SABVL | | | |
| | SABVR | | | |
| SIA | SIADL | | | |
| | SIADR | | | |
| | SIAVL | | | |
| | SIAVR | | | |
| SIB | SIBDL | | | |
| | SIBDR | | | |
| | SIBVL | | | |
| | SIBVR | | | |
| SMB | SMBDL | | | |
| | SMBDR | | | |
| | SMBVL | | | |
| | SMBVR | | | |
| SMD | SMDDL | | | |
| | SMDDR | | | |
| | SMDVL | | | |
| | SMDVR | | | |
| URA | URADL | | | |
| | URADR | | | |
| | URAVL | | | |
| | URAVR | | | |
| URB | URBL | | | |
| | URBR | | | |
| URX | URXL | | | |
| | URXR | | | |
| URY | URYDL | | | |
| | URYDR | | | |
| | URYVL | | | |
| | URYVR | | | |

Neuron fate
transformed to Hyp
transformed to CD
too late / ambigious

Glutamatergic (EAT-4(+))
Cholinergic (CHO-1(+))
UNC-42(+)
TTX-3(+)
UNC-86(+)
UNC-3(+)

122 neuron born
69 to Hypo
9 to CD
21 undetected

Development • Supplementary information

**Table S3. Tabular summary of cell fate transformation in *hlh-2*$^{m/z}$ mutant animals**. Like Table S2, this table extracts information from the lineage diagram of *hlh-2*$^{m/z}$ mutant animals (**Fig.2**), here with a focus on the name of blast cells transformed to alternative identities.

| Transformation to cell death | Fate in N2 | Fate in *hlh2*$^{m/z}$ |
|---|---|---|
| AB pl/rpaapaap | AVB | CD |
| AB plppaappp | DB6 | CD |
| AB prppappppa/p | DD4/ DD6 | CD |
| AB pl/rpaapaaa | RIV | CD |
| ABalapaapaa/ ABalaapppaa | RIA | CD |

| Transformation from cell death | Fate in N2 | Fate in *hlh2*$^{m/z}$ |
|---|---|---|
| AB prppaaap | CD | Neuron |
| AB alppaapa | CD | Neuron |
| AB alaapappa | CD | Neuron |
| AB alaapapp | CD | Neuron |
| AB prppaaap | CD | Neuron |
| AB praaapaa | CD | Hypo |
| AB praaapap | CD | Hypo |
| AB prapaaaa | CD | Hypo |
| AB plapaaaa | CD | Hypo |
| AB plapappp | CD | Hypo |
| ABprapppaap | CD | Hypo |
| ABprapppap | CD | Hypo |
| ABplapppaap | CD | Hypo |
| ABpapppapa | CD | Hypo |
| ABpaaaapp | CD | Hypo |
| ABpraaapaap | CD | Hypo |
| ABpraaapapp | CD | Hypo |
| ABalpppaapp | CD | Hypo |
| ABalppppaap | CD | Hypo |
| ABalppppapp | CD | Hypo |
| ABalppaaap | CD | Hypo |
| ABalapapaa | CD | Hypo |
| Caapap | CD | Hypo |
| AB praaaapp | CD | Hypo |
| ABalppaaaa | CD | Mutant |
| ABalppaapa | CD | Mutant |
| ABalppaapa | CD | Mutant |

| Transformation in non-neuronal | Fate in N2 | Fate in *hlh2*$^{m/z}$ |
|---|---|---|
| | ILsoDR/DL | Hypo |
| | OLQshDL | Hypo |
| | CEPshDR | Hypo |
| | Tail spike | CD |
| | Hyp10 | CD |
| MSppaaaaa/p | Muscle | CD |
| | GLRL/VR | Mutant |

| Transformation to Hypodermal cells | Fate in N2 | Fate in *hlh2*$^{m/z}$ |
|---|---|---|
| AB pl/rapaaaapp | ADA | Hypo |
| AB pl/rapaaaapa | ADE | Hypo |
| AB al/rppppaa | ADF | Hypo |
| AB alppppaad / AB praaapaad | ADL | Hypo |
| AB alpppapav / AB praaaapav | AFD | Hypo |
| AB pl/raapappa | AIB | Hypo |
| AB pl/rapaaapav | AIZ | Hypo |
| AB alapppaaa | ALA | Hypo |
| AB arppa/pappa | ALM | Hypo |
| AB pl/rapappppap | ALN | Hypo |
| AB alpppppaa / AB praaapppaa | ASE | Hypo |
| AB pl/raapapap | ASG | Hypo |
| AB pl/raapapppa | ASI | Hypo |
| AB alpppppppa / AB praaappppa | ASJ | Hypo |
| AB alppppppp / AB praaappppp | AUA | Hypo |
| AB alapa/ppppa | AVJ | Hypo |
| AB pl/raapapaa | AWA | Hypo |
| AB alpppppap / AB praaappap | AWB | Hypo |
| AB arppa/pappp | BDU | Hypo |
| AB alapaaapa / AB alappappa | CAN | Hypo |
| C aapaa | DVC | Hypo |
| AB pl/rapaaapad | FLP | Hypo |
| AB pl/rapppappa | HSN | Hypo |
| AB alppppapaa / AB praaapapaa | OLL | Hypo |
| AB pl/rapppappp | PHB | Hypo |
| AB pl/rapappppaa | PLM | Hypo |
| AB pl/rapppaaa | PVQ | Hypo |
| C aappa | PVR | Hypo |
| AB alappaapa | RID | Hypo |
| AB pl/rapaaapp | RMG | Hypo |

**Asymmetric transformation to Hypodermal cells**

| | Fate in N2 | Fate in *hlh2*$^{m/z}$ |
|---|---|---|
| AB praaaappa | ASKR | Hypo |
| AB alappapaa | AVHR | Hypo |
| AB alapa/pppaaa | IL1D | Hypo |
| AB alapa/pppap | IL2D | Hypo |
| AB alapppapaa | OLQDR | Hypo |
| AB prppaapap | RIMR | Hypo |
| AB alpppapad/ AB praaaapad | RMDL/R | Hypo |
| AB alapppaap | RMED | Hypo |
| AB arpapaaaa | URADR | Hypo |
| AB alapppapp | URYDR | Hypo |

Development • Supplementary information
